# Magnitude of Reduction and Speed of Remission of Suicidality for Low Amplitude Seizure Therapy (LAP-ST) Compared to Standard Right Unilateral Electroconvulsive Therapy: A Pilot Double-Blinded Randomized Clinical Trial

**DOI:** 10.3390/brainsci9050099

**Published:** 2019-04-29

**Authors:** Nagy A. Youssef, Dheeraj Ravilla, Cherishma Patel, Mark Yassa, Ramses Sadek, Li Fang Zhang, Laryssa McCloud, William V. McCall, Peter B. Rosenquist

**Affiliations:** 1 Department of Psychiatry and Health Behavior, Medical College of Georgia at Augusta University, Augusta, GA 30912, USA; LRAVILLA@augusta.edu (D.R.); CHEPATEL@augusta.edu (C.P.); MYASSA@augusta.edu (M.Y.); LMCCLOUD@augusta.edu (L.M.); WMCCALL@augusta.edu (W.V.M.); PROSENQUIST@augusta.edu (P.B.R.); 2 Office of Academic Affairs, Medical College of Georgia at Augusta University, Augusta, GA 30912, USA; 3 Department of Population Health Science, Medical College of Georgia at Augusta University, Augusta, GA 30912, USA; RSADEK@augusta.edu (R.S.); LIZHANG@augusta.edu (L.F.Z.); 4 Georgia Cancer Center, Medical College of Georgia at Augusta University, Augusta, GA 30912, USA

**Keywords:** electroconvulsive therapy, low amplitude seizure therapy, seizure therapy, focal ECT, precision low amplitude seizure therapy, treatment-resistant depression, mood disorders, Randomized Clinical Trial

## Abstract

**Background**: Although treatment guidelines support use of electroconvulsive therapy (ECT) for acute suicidality, it is associated with cognitive side effects. The effect of Low Amplitude Seizure Therapy (LAP-ST) on suicidality is unknown. Our prior precision LAP-ST (pLAP-ST) performing titrating in the current domain has provided initial proof of concept data in humans of its advantage in terms of reduction of cognitive side effects. The aims of this report are to: 1) compare LAP-ST (at 500mA) versus standard Right Unilateral (RUL) ECT (at 900 mA) in terms of magnitude of remission of suicidality in a randomized allocation and 2) compare the speed of remission of suicidality between LAP-ST versus RUL ECT. **Methods**: Patients were randomized to either LAP-ST or RUL ECT. The scores pertaining to the suicidal ideation (SI) item on the Montgomery-Åsberg Depression Rating Scale (MADRS) were analyzed using descriptive analysis and no confirmatory statistical analysis was performed due to a priori sample size limitations for this pilot study. SI item remission was defined as 2 or below on this item. **Results**: Eleven patients with major depressive episode (MDE) of mainly unipolar or bipolar disorders signed consent. Of these, 7 were eligible and were randomized and included in the analysis; all were actively suicidal at baseline (suicide item above 2), except 1 patient who had suicide item at 2 in the RUL ECT group. Suicidality remitted on average by session 3 and remission occurred for all patients by session 4. The SI mean score improvement from baseline to endpoint for LAP-ST was 5.1 and for RUL ECT was 3.0. **Conclusions**: LAP-ST has larger effect size and speed of remission of suicidality compared to standard RUL ECT. Future studies are warranted for replicating these findings. (ClinicalTrials.gov ID: NCT02583490).

## 1. Introduction

Among the leading causes of death, suicide is one of only three causes that are increasing [1,2]. In 2016, about 45,000 people ≥10 years of age died of suicide (15.6/100,000) in the United States [1]. Suicide rates increased from 1999 to 2015 [3,4], with adults aged 45–64 having the highest rate of increase (from 13.2 per 100,000 persons (1999) to 19.2 per 100,000 (2016)) [1,4]. Suicide has increased among both sexes and all racial/ethnic groups [3,4].

In addition, major depressive episode (MDE)—whether being part of major depressive disorder (MDD), bipolar disorder (BD) or schizoaffective disorder—is a major risk for suicide [5,6]. Of those with MDE, up to a third present with treatment-resistant depression (TRD) [7,8]. Patients with TRD tend to have higher risk of suicide [9], with higher number of prior suicide attempts, suicidal thoughts and lower quality of life [10,11].

Not only does electroconvulsive therapy (ECT) has anti-suicidal effect but also has been shown in the PRIDE study (a large multicenter clinical trial) to improve quality of life in depressed patients [10]. ECT is underutilized in the treatment of both TRD and suicidality [12]. This may be attributed, at least in part, to the fear of cognitive side effects of ECT. Cognitive side effects of ECT may also partially contribute to stigma of ECT as well as reduce access to ECT for those patients who need it most [13,14]. A retrospective study of the National Suicide Prevention Project in Finland found that among all (*n* = 1397) suicides in a 12-month period, only 2 patients (0.14%) underwent ECT compared with other forms of treatments [15].

There is limited literature on the effect of ECT on suicidality. However, ECT is sometimes used clinically when quick and reliable relief of suicidal intent and reduction of suicide risk is needed, with high rates of success [12]. On the other hand, cognitive (including memory) side effects of ECT can be a major problem to patients and families during or after ECT course or can be a deterrent to starting an effective course of ECT altogether. This might explain, at least in part, the delayed use associated with ECT [16]. Thus, probably more lives could be saved if the worry about memory and cognitive problems can be abated or minimized.

Fortunately, evidence from our prior precision LAP-ST (pLAP-ST) proof of concept open-label study has provided initial evidence of the advantage of LAP-ST in terms of cognitive side effects in humans [17]. After initial pilot titrations in the current domain to find the most appropriate range and titration combinations for the proof of concept study [18], we proceeded with enrollment for the pLAP-ST study in 2013 where we did titration in the current domain that ranged from 500mA to a maximum of 600mA. This was the first study in humans to include a full course of LAP-ST and included 22 patients who were clinically indicated to undergo ECT. The study showed that pLAP-ST course was feasible in humans. All patients had a seizure in the first session. While both pulse width and duration of stimulus were fixed at 0.3 ms and duration of 8s respectively, we titrated the current domain from 500mA to 600mA to tailor the current dose more precisely. A maximum amplitude of up to 600mA was allowed in this study. Successively, if needed for seizure induction, the frequency of the stimulus was titrated up, starting with 20 Hz. Subsequent sessions charge was 6 times that of the seizure threshold of the first session. Participants had a quick orientation with median time to reorientation (TRO) of 4.5 min (and mean of 6.6 min), a strong predictor of memory and cognitive side effects. The TRO was much less than historical data of mean TRO in studies for both bitemporal and standard RUL ECT using standard current in the 800–900mA range [17,18,19], suggesting less cognitive and memory side effects in this pLAP-ST study. Treatment was efficacious for both depressive and psychotic symptoms.

However, both the effect of LAP-ST on suicidality and the speed of remission of suicidality using LAP-ST is still unknown. The present study is a randomized pilot clinical trial initiated in 2015 (ClinicalTrials.gov ID: NCT02583490) to assess several outcomes. The aims of this report are to: 1) compare LAP-ST (at 500mA) versus standard Right Unilateral (RUL) ECT (at 900mA) in terms of magnitude of remission of suicidality and 2) compare the speed of remission of suicidality between LAP-ST versus RUL ECT. We hypothesized that there will be a similar improvement in magnitude and in the speed of remission of suicidality on the suicidality item of the Montgomery-Åsberg Depression Rating Scale (MADRS) for LAP-ST compared to RUL ECT [20,21].

## 2. Methods

### 2.1. Participants

The Institutional Review Board of the Medical College of Georgia (MCG) at Augusta University approved this study. After full explanation of the study, including potential risks and benefits, written informed consent was obtained from participants prior to enrollment in the study. Study participants were recruited from the ECT program at MCG including patients from inpatient, outpatient, consultation-liaison services, and private practice referrals. Participants who met the inclusion criteria of the study started baseline assessments.

### 2.2. Eligibility

Inclusion criteria for participation in the study were as follows: (1) Patients in whom ECT therapy is clinically indicated, (2) Male or female patients over 20 years of age, (3) Current Diagnostic and Statistical Manual of Mental Disorders, 4th Edition (DSM-IV) criteria for major depressive episode (MDE) of major depressive or bipolar disorders or schizoaffective disorder, (4) Montgomery-Åsberg Depression Rating Scale (MADRS) of 20 or higher at baseline, (5) Use of effective method of birth control for women of child-bearing capacity, (6) Medically stable, (7) No anticipated need to alter psychotropic medications for the duration of the study and (8) Ability of patient to fully participate in the informed consent process.

Exclusion criteria included the following: (1) Unstable or serious medical condition that substantially increases risks of ECT or of cognitive impairment, (2) Substance use disorders within 1 week of randomization, (3) History of neurological disorder including: epilepsy, stroke, brain surgery, metal in the head, history of known structural brain lesion that is deemed to affect cognition or safe ECT treatment, (4) Vagus Nerve Stimulator implanted, (5) Female patients who were pregnant or planned to be pregnant during the study; breast-feeding, (6) Implanted devices that make ECT unsafe or a skull defect, (7) Significant cognitive impairment as judged clinically by one of the study psychiatrists or the principal investigator (PI).

### 2.3. Assessments

Participants were diagnosed according to DSM IV criteria using the Structured Clinical Interview for DSM IV. The analysis of this report included baseline assessments of the suicide item of Montgomery-Åsberg Depression Rating Scale (MADRS) (item 10) [22], prior to each ECT session, as well as 1-2 days after the end of the ECT course. All baseline and endpoints assessments were done by the principal investigator (NY) who was blinded to the treatment allocations. Secondary assessments during treatment sessions were done either by the PI or other trained blinded assessors (student research assistants, DR, CP, MY). Remission of suicidality was defined as 2 or below on the suicidality item (0 to 6 score) [23]. The study was double-blinded where the PI, clinical raters and patients were masked to the treatment conditions.

### 2.4. Procedure

Patients received the standard clinical pre-ECT medical and laboratory workup as indicated by the treating clinicians for safe delivery of ECT and anesthetic procedures. Medication and anesthesia adjustments were used according to the discretion of the treating psychiatrist and ECT-trained anesthesiologist as deemed clinically necessary according to the standard of care. A bite-block was inserted prior to seizure elicitation to protect the teeth.

All other standard safety procedures for ECT were done as per the American Psychiatric Association ECT Guideline [24]. Standard ECT or LAP-ST sessions were given three times per week.

### 2.5. Low Amplitude Seizure Therapy (LAP-ST) or Standard RUL ECT

LAP-ST was performed using a MECTA Spectrum 5000Q device and Right Unilateral (RUL) electrode placement in both arms of the study. Seizure Threshold (ST) was estimated by empiric titration in the first session of each participant. For seizure titration in the first session: Stimulus parameters were fixed at pulse width of 0.3 ms and current amplitude of 900mA in the standard arm and pulse width of 0.3 ms and current amplitude of initially 500mA in the low amplitude arm. ST was then determined as presented in Table 2 by the titration schedule for the initial titration session. The subsequent treatment sessions were dosed at 6 times the charge of the seizure threshold (unless titration has to utilize step 4, in this case less than 6-times the seizure threshold was used due to device limitation). All electric parameters for both arms of the study are presented in detail in Table 2.

### 2.6. Anesthesia

Anesthesia was administered by anesthesiologists with specific training and experience in ECT. Briefly, blood pressure, pulse, ECG and pulse oximetry and capnography were monitored prior to anesthetic induction and continuously during the procedure. Standardized anesthesia procedures were used including induction with methohexital (~1 mg/kg) in general unless clinically indicated to use other types of anesthesia. The standard muscle relaxation used was succinylcholine (~0.75 mg/kg). The protocol also allowed alternate inducing agent (such as etomidate (~0.3 mg/kg) or alternate muscle relaxant was permitted only if clinically indicated. Patients were ventilated with 100% oxygen from anesthetic administration until return of spontaneous respirations. Seizure expression was monitored by bilateral fronto-mastoid EEG and inspection of the cuffed ankle to record motor manifestations. Both the duration of the EEG and motor seizure durations were recorded in seconds.

### 2.7. Electroencephalography (EEG) Acquisition and Ictal Monitoring

EEG seizure duration was monitored using two EEG channels (fronto-mastoid electrodes). The duration of the seizure in the brain was determined by the EEG recording. Motor manifestations of seizures were monitored using the cuff technique. Motor manifestations were observed with inspection of the cuffed ankle and the body for motor seizures.

### 2.8. Data Analysis

The data were visualized and assessed for normality (i.e., normal distribution). Based on the normality of the data or lack of normality, it was decided *a priori* to either use parametric or non-parametric tests, respectively. There was no major deviation from normal distribution of the data and sample characteristics were reported as mean and SD for continuous variables and as percentages for discontinuous variables.

The scores of the suicidal ideation (SI) item on the MADRS were analyzed. SI item remission was defined as 2 or below [23]. The percentage of patients unremitted for SI were calculated. SPSS version 23, (Inc., Chicago, IL, USA) was used for the statistical analyses.

## 3. Results

### 3.1. Demographic and Clinical Characteristics

**Figure 1:**
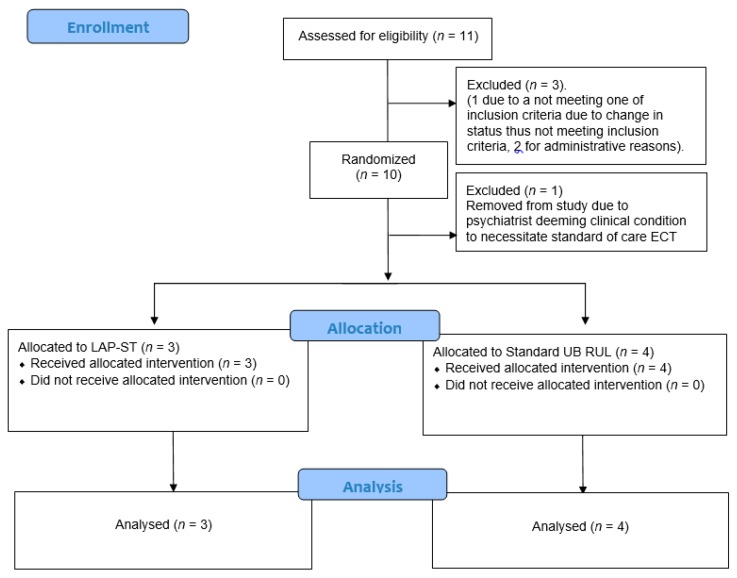
CONSORT Diagram.

Eleven patients gave their informed consent. Three patients were not randomized (one did not meet one of inclusion criteria due to change in status after consenting and two for administrative reasons). One patient was discontinued by the treating psychiatrist prior to the treatment session due to deeming clinical condition to necessitate standard of care ECT in a non-randomized fashion. Thus, seven patients were included in the intention to treat analysis (see Figure 1 “Consort diagram”). These seven patients completed one or more treatment sessions (session in which ECT charge was given at therapeutic levels). Mean, median and range of ages from each group as well as other demographic characteristics are presented in Table 1. Frequencies and percentages are reported for the categorized variable, such as gender, race, education and diagnosis.

### 3.2. Treatment Characteristics, Parameters and Seizure Duration

The titration algorithm for the electric parameters for the first titration session as well as for subsequent treatment sessions are presented by treatment group in Table 2. Mean electrical parameters of titration in session 1 and subsequent treatment sessions of the RUL ECT and LAP-ST groups, as well as seizure duration for the standard RUL ECT group and for LAP-ST group are described in Table 3. We used 6-times seizure threshold in subsequent session after the titration session (except for 1 patient in LAP-ST group where lower charge was used based on clinical judgment by the ECT psychiatrists).

The mean number of sessions for the LAP-ST group was 6.3 and the median was 6 (range for completers was 6–9). The mean number of sessions for the RUL ECT group was 6 and the median was 6.5 (range for completers was 5–8).

### 3.3. Suicidality Remission

**Figure 2:**
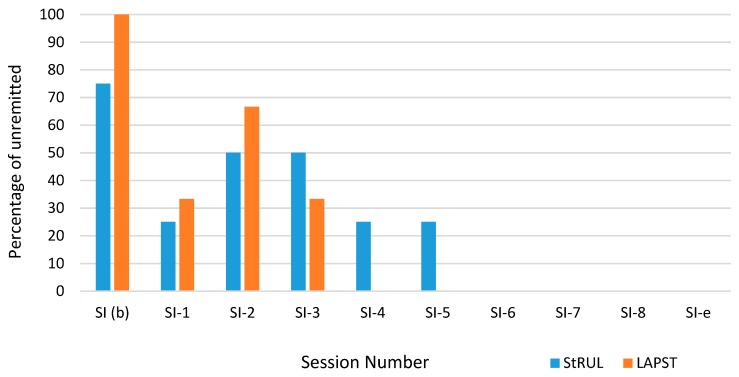
Percentages of Unremitted Suicidal Ideation each Session for RUL ECT vs LAP-ST.

**Figure 3:**
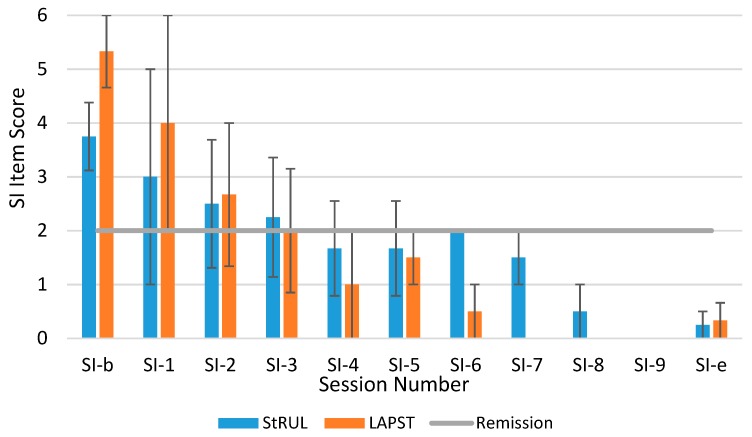
Means of suicidal ideation (SI) Item Score by Treatment Group in Each Session.

All patients had active suicidality at baseline (suicide item above 2), except 1 patient who had suicide item at 2 in the RUL ECT group. The percentages of patients with unremitted suicidality by treatment session and by study group are presented in Figure 2. In LAP-ST, suicidality remitted by session 3 on average and remission occurred for 100% of patients in both groups by session 4 (see Figure 2 and Figure 3) (despite the fact that baseline mean suicidality for LAP-ST was higher than for standard RUL ECT group). The average scores pertaining to the SI item and their standard errors for two treatment groups in each session are presented in Figure 3.

The SI mean score improvement from baseline to endpoint for LAP-ST was 5.1 and for RUL ECT was 3.0. The magnitude of reduction of the MADRS suicidality item in each session for RUL ECT versus LAP-ST is presented in Figure 3.

**Figure 4:**
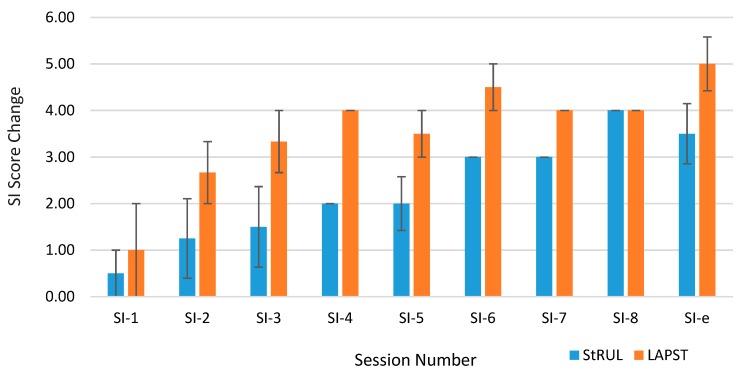
Suicidal Ideation Item Score of Average Change from Baseline at Each Session.

The findings indicated improvement in suicidality between baseline and endpoint. The average change (improvement) of the suicidality item score from baseline by group at each session is presented in the Bar Chart in Figure 4.

## 4. Discussion

The aims of this report were to examine in this pilot randomized study, whether the magnitude of reducing of SI and speed of SI remission will be comparable for both standard RUL ECT and LAP-ST. We found that in this study suicidality remitted by session 3 on average for LAP-ST and remission occurred for all patients by session 4. In addition, the effect size for the SI change score (reduction in SI) for the LAP-ST was greater than that of the standard RUL ECT group.

Rapid onset and anti-suicidal effects have been observed with both bitemporal (BT) and right unilateral ECT [25] but never examined in studies utilizing low amplitude modality. This study adds to the limited research on the speed of response of SI using ECT/seizure therapy.

Our study used “remission” of suicidality as an item score of 2 or lower in MADRS (0 to 6 score for suicidality item) and above 2 to represent active suicidality, whereas Fink [26] and other studies use “resolution” or absence of suicidality as an item score of 0 on the Hamilton Rating Scale for Depression 24-item (0- to 4 on the suicidality item. Other studies used a cutoff of below 4 on the suicidality item of the MADRS as an indication of "without recent serious suicidal ideation (MDD-non-suicide)" [23].

Even with the different scales, some comparisons can be made between our study and the Fink meta-analysis. In a sub analysis, we defined “resolution” as 0 out of 6 on the MADRS suicidality item. In the LAP-ST group, 33.3% reached resolution (zero score) after 1 ECT session, 66.7% after 3, 6 and 9 sessions. In the RUL ECT group, 33.3% reached resolution after 1 ECT session, 33.3% after 3 sessions, 66.7% after 6 sessions and 9 sessions. The results of this study demonstrate comparable rates of progression toward remission of suicidality in each treatment group.

Limitations of this study include the small sample size and that suicidality was not part of the inclusion criteria. Although suicidality was not part of the inclusion criteria, all patients at baseline had suicidality item score above zero. Another limitation is that the long-term anti-suicidal effects of ECT or LAP-ST were not examined in this study. However, prior data from continuation treatments have similar 6-month remission rates after successful courses of ECT (ECT or combined nortriptyline and lithium) in depressed patients. [26].

Nonetheless, strengths of this pilot study include the double-blinded and the randomized design. It is thus the first randomized double-blinded study of LAP-ST in humans. The results of this study are promising, suggesting that both LAP-ST and standard RUL ECT are associated with rapid and similar remission of suicidality. The results should be replicated in large clinical trials with similar design with longer follow-up period to show long-term effects of LAP-ST on suicidality. This could potentially help move LAP-ST towards the clinical domain as a viable option of treatment for acute suicidal patients with less cognitive side effects [17].

## 5. Conclusions

This was the first LAP-ST study showing reduction in SI with LAP-ST. The effect size of reduction for the suicide scores from baseline was 5.1 for LAP-ST group, while it was 3.0 for the standard RUL ECT group. This adds support to our prior pLAP-ST proof of concept clinical trial [17] that LAP-ST can produce effective therapeutic outcomes.

## Figures and Tables

**Table 1:** Demographic and Clinical Characteristics of the Total Sample of Patients.

Variable	Stat	Total Sample (*n* = 11)	Standard RUL (*n* = 4)	LAP-ST (*n* = 3)	Dropouts (*n* = 4)
**Age**	Mean (SD)	40.64 (13.81)	40.25 (17.39)	42.00 (16.09)	40 (12.57)
	Median	44	39	47	37.5
	Range	24–59	24–59	24–55	29–56
**Female**	N (%)	6 (54.55)	3 (75.00)	0 (0.00)	3 (75.00)
**Race**					
Black or African American	N (%)	2 (18.18)	0 (0.00)	1 (33.33)	1 (25.00)
White	N (%)	8 (72.73)	4 (100.00)	2 (66.67)	2 (50.00)
Other	N (%)	1 (9.09)	0 (0.00)	0 (0.00)	1 (25.00)
**Education**					
High School graduate-diploma or GED	N (%)	2 (18.18)	0 (0.00)	1 (33.33)	1 (25.00)
Some College	N (%)	4 (36.36)	2 (50.00)	1 (33.33)	1 (25.00)
College graduate	N (%)	4 (36.36)	1 (25.00)	1 (33.33)	2 (50.00)
Master’s Degree	N (%)	1 (9.09)	1 (25.00)	0 (0.00)	0 (0.00)
**Diagnosis**					
Major Depressive Disorder	N (%)	4 (36.36)	2 (50.00)	1 (33.33)	1 (25.00)
Bipolar Type I	N (%)	5 (45.45)	1 (25.00)	2 (66.67)	2 (50.00)
Bipolar Type II	N (%)	1 (9.09)	0 (0.00)	0 (0.00)	1 (25.00)
Schizoaffective/bipolar type	N (%)	1 (9.09)	1 (25.00)	0 (0.00)	0 (0.00)

**Table 2:** The Titration Algorithm for the Electric Parameters for the First Titration Session and Subsequent Treatment Sessions by Treatment Group.

		Low Amplitude Seizure Therapy (LAP-ST)		Standard Right Unilateral (RUL) ECT	
		Titration Session	6 × ST * (500%)	Titration Session	6 × ST * (500%)
Stimulus 1	PW	0.3 ms	0.3 ms	0.3 ms	0.3 ms
	Frequency	20 Hz	25 Hz	20 Hz	20 Hz
	Duration	2 s	8 s	1 s	6 s
	Current	500 mA	500 mA	900 mA	900 mA
	Charge	12 mC	60 mC	10.8 mC	64.8 mC
Stimulus 2	PW	0.3 ms	0.3 ms	0.3 ms	0.3 ms
	Frequency	20 Hz	60 Hz	20 Hz	35 Hz
	Duration	4 s	8 s	2 s	7.5 s
	Current	500 mA	500 mA	900 mA	900 mA
	Charge	24 mC	144 mC	21.6 mC	141.75 mC
Stimulus 3	PW	0.3 ms	0.3 ms	0.3 ms	0.3 ms
	Frequency	20 Hz	120 Hz	20 Hz	65 Hz
	Duration	8 s	8 s	4.5 s	8 s
	Current	500 mA	500 mA	900 mA	900 mA
	Charge	48.0 mC	288 mC	48.6 mC	280.8 mC
Stimulus 4	PW	0.5 ms	0.5 ms	0.3 ms	0.34 ms
	Frequency	60 Hz	120 Hz	25 Hz	115 Hz
	Duration	8 s	8 s	8 s	8 s
	Current	600 mA	600 mA	900 mA	900 mA
	Charge	108.0 mC	576 mC	172.0 mC	563.0 mC

* Six times seizure threshold were used in subsequent treatment sessions unless limited by the maximum output of the device.

**Table 3:** Mean Electric Parameters of Titration Session and Subsequent Sessions by Treatment Group.

Parameters	Standard RUL (*n* = 4)		LAP-ST (*n* = 3)	
	1st Session	Subsequent Sessions	1st Session	Subsequent Session
Charge (mC), mean (SD), mean (SD)	18.9 (5.4)	122.5 (38.48)	73.33 (72.04) *	288.0 (173.1) *
Current (mA), mean (SD), mean (SD)	900 (0)	900 (0)	500 (0)	500 (0)
Pulse Width (Sec), mean (SD)	0.3 (0)	0.3 (0)	0.53 (0.40)	0.60 (0.36)
Frequency (Hz), mean (SD)	23.75 (7.5)	31.25 (7.5)	35 (25.98)	70 (45.83)
Motor Seizure (Sec), mean (SD)	56.25 (24.30)	21.25 (8.10)	27.67 (13.50)	36.00 (12.53)
EEG Seizure (Sec), mean (SD)	81.5 (49.22)	42.0 (5.72)	44.67 (36.14)	51.33 (21.03)

* Six-times seizure threshold were used in subsequent treatment sessions unless limited by the maximum output of the device.
